# Photobiomodulation therapy on the palliative care of temporomandibular disorder and orofacial/cervical skull pain: study protocol for a randomized controlled clinical trial

**DOI:** 10.1186/s13063-019-3294-7

**Published:** 2019-04-06

**Authors:** Fernando Rodrigues Carvalho, Rafael Queiroz Barros, Alyne Simões Gonçalves, Patrícia Moreira Freitas

**Affiliations:** 1 Special Laboratory of Lasers in Dentistry (LELO), School of Dentistry of the University of São Paulo (USP), São Paulo, Brazil; 2 Department of Biomaterials and Oral Biology, School of Dentistry, University of São Paulo (USP), São Paulo, Brazil; 3 Department of Restorative Dentistry, Special Laboratory of Lasers in Dentistry, School of Dentistry, University of São Paulo (USP), Avenida Professor Lineu Prestes, 2227, São Paulo, 05508-000 Brazil

**Keywords:** Temporomandibular disorder, Lasers, Photobiomodulation, Laser therapy

## Abstract

**Background:**

Temporomandibular disorder (TMD) is the most common cause of orofacial and cervical skull pain and is considered to be a public health problem, affecting 5% to 12% of the world population. TMD is multifactorial and there are several types of treatment, with the conservative types being indicated more often as they are less aggressive and reversible. The main aim of these treatments is to relieve symptoms, reduce of pain, and restore orofacial and cervical skull functions. Photobiomodulation therapy (PBMT), a noninvasive therapy, is an option for the management of musculoskeletal disorders due to its analgesic, anti-inflammatory, and regenerative effects.

**Methods:**

The aim of the proposed study is to verify whether PBMT is effective for use in palliative care of TMD and orofacial and cervical skull pain. A randomized, triple-blinded, placebo-controlled clinical trial is proposed. This study will involve 200 adult participants (over 18 years of age) who will be randomly divided into two groups (*n* = 100): Group 1, active treatment (PBMT); and Group 2, placebo. Participants will be subjected to three sessions of PBMT or placebo and will be evaluated using the research diagnostic criteria (RDC) for TMD. Pain level (measured by a visual analog scale (VAS)), mandibular movements (measured by ruler and caliper), quality of life (measured by the Oral Health Impact Profile (OHIP)-14), and quality of sleep (measured by the Epworth scale) will be recorded. This study is being conducted at the Special Laboratory of Lasers in Dentistry (LELO) of the School of Dentistry of the University of Sao Paulo (USP).

**Discussion:**

This study will verify whether PBMT is effective in reducing TMD and orofacial and cervical skull pain. PBMT may be an option for the management of musculoskeletal disorders due to its analgesic, anti-inflammatory, and regenerative effects, in addition to being a noninvasive technique.

**Trial registration:**

Registro Brasileiro de Ensaios Clínicos, RBR-9b6mnj. Registered on 27 March 2018.

**Trial registry name:** Laser de baixa potência no cuidado paliativo da disfunção temporomandibular e dor crânio orofacial e cervical.

**Ethics committee:** #1774930 approved on 14 October 2016.

**Electronic supplementary material:**

The online version of this article (10.1186/s13063-019-3294-7) contains supplementary material, which is available to authorized users.

## Background

Temporomandibular disorder (TMD) is a term used to describe a number of clinical problems involving the temporomandibular joint and masticatory muscles and other associated structures [1].

TMD, the most common cause of orofacial and cervical skull pain, is considered a public health problem as it affects 5% to 12% of the world population. Ranked as the second major cause of skeletal muscle pain, its main consequences are impairment relative to performing daily activities, psychosocial functioning, and quality of life [2].

The TMD etiology is multifactorial and is commonly related to trauma, neoplasia, stress and anxiety, occlusal interferences, poorly positioned teeth, bruxism, tooth/teeth loss, poor relationship of bone bases, parafunctional movements, deleterious habits, and intrinsic temporomandibular joint problems, which may or may not appear in combination [3–5].

The most common symptoms are articular and auricular pain, joint noises (crackling and/or clicking), tinnitus, headache, muscle fatigue in the orofacial and cervical skull region, and limitation of mandibular movements (opening and laterality) [6–9].

There are several types of treatments proposed for TMD, with the conservative types being more indicated because they are less aggressive and reversible. The main aims of these treatments are to relieve symptoms, reduce pain, and re-establish orofacial and cervical skull functions [10].

Photobiomodulation therapy (PBMT) is an option for the management of musculoskeletal disorders due to its analgesic, anti-inflammatory, and regenerative effects, and because it is a noninvasive technique [11–13]. Several authors have previously reported an improvement in symptoms caused by TMD when considering PBMT. Carvalho et al. [13] conducted a clinical study in which 74 subjects diagnosed with TMD were submitted to PBMT and concluded that irradiation with red and infrared lasers was effective in reducing muscular pain. Venezian et al. [14] evaluated 48 subjects and reported a reduction in TMD symptoms after PBMT. The authors concluded that pain related to both the masseter and temporal muscles (anterior beam) decreased after laser irradiation. Khalighi et al. [15] conducted a study comparing the use of a nonsteroidal anti-inflammatory drug (NSAID) with PBMT and the results showed that the PBMT was more effective than the NSAID in reducing pain intensity and increasing mouth opening amplitude. Salmos-Brito et al. [16] investigated 58 patients with myogenic pain caused by acute and chronic mandibular temporomandibular dysfunction and concluded that all subjects presented positive results with PBMT; for acute myogenic pain, the results were better than those found for chronic myogenic pain.

Although there are many studies that report the effects of PBMT on TMD, there is still no consensus about the potential of PBMT using a low-power laser on decreasing orofacial/cervical skull pain caused by TMD.

### Objectives

The present clinical trial aims to verify: 1) whether PBMT using a low-power laser is effective in the palliative care of TMD and orofacial/cervical skull pain by decreasing pain in masticatory muscles and orofacial/cervical regions; 2) the duration of effects produced by PBMT; 3) whether PBMT is effective in reducing “clicking” and/or “crackling”; 4) whether PBMT improves patients’ quality of life; and 5) whether PBMT improves patients’ perception of sleep quality.

### Hypotheses

The hypotheses of the present clinical trial are: 1) PBMT is effective in the palliative care of TMD and orofacial/cervical skull pain by decreasing pain levels in masticatory muscles and orofacial/cervical regions; 2) the effects of PBMT do not last up to 7 days; 3) “clicking” and/or “crackling” decreases after PBMT; 4) PBMT improves patient quality of life; and 5) PBMT therapy improves patient quality of sleep.

### Expected results

The PBMT is expected to be effective in the palliative care of TMD and orofacial/cervical skull pain by decreasing pain in masticatory muscles and orofacial/cervical regions and improving oral functions, quality of life, and perception of sleep quality. The positive effects of the therapy are expected to be able to last for up to 7 days and that the clicking or cracking will decrease after PBMT.

## Methods/design

### Study design

**Fig. 1:**
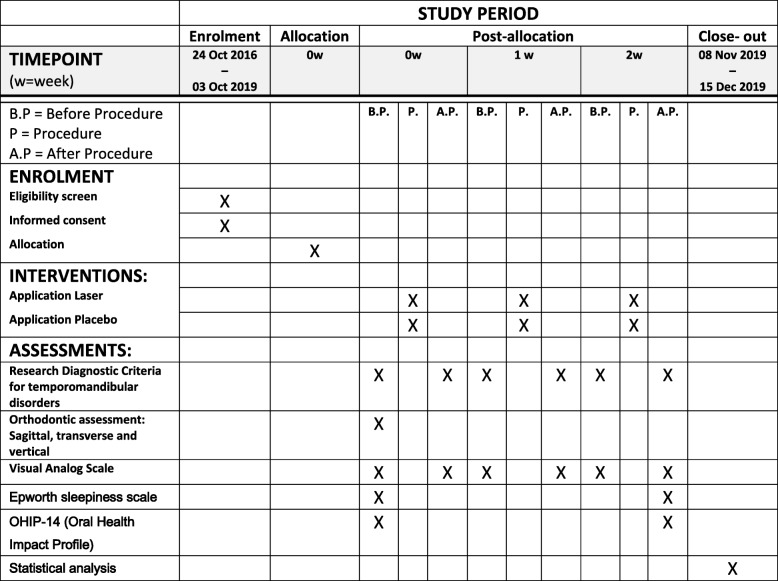
Overall schedule of enrollment, interventions, and assessment, in accordance with the SPIRIT statement

This protocol is presented in accordance with the SPIRIT 2013 (Standard Protocol Items: Recommendations for Interventional Trials) Statement (see Additional file 1 for the Spirit Checklist and Fig. 1 for the trial schedule of enrollment, interventions, and assessment in accordance with the recommended Spirit figure).

A randomized, triple-blinded, placebo-controlled clinical trial, with two parallel groups, approved by the Research Ethics Committee of the University of São Paulo (USP), Brazil (protocol no. 1774930), will be conducted at the Special Laboratory of Lasers in Dentistry (LELO). All potential participants will be informed about all details of the study (number of sessions, procedure, risks, and discomforts). All participants who agree to participate will sign a Term of Free and Informed Consent, as required by the Brazilian National Board Health.

The researchers (FRC and RQB) in charge of irradiating the participants, the participants, and the statistician will be blinded to the treatment groups (active or placebo). The other researcher (PMF) who is not involved in laser application will be in charge of setting up the equipment for each experimental group: active and placebo (without emission of laser light).

### Randomization

The participants will be randomly allocated to one of the two groups (*n* = 100): active treatment (PBMT) or placebo. Randomization will be performed in blocks of 50 participants, which means that 50 opaque sealed envelopes (25 envelopes for the active intervention group and 25 envelopes for the placebo group) will be mixed and then numbered sequentially. This procedure will be repeated four times. A researcher (PMF) not involved in the clinical steps will be in charge of randomization/allocation of patients to the groups.

### Participants

#### Inclusion criteria

Adults of all ages will be included, both males and females, regardless of race or social class, with a main complaint of pain in the temporomandibular joint (TMJ) region and/or orofacial/cervical skull region, with or without limitation in mouth opening. Participants will be screened at the clinic of TMD of the School of Dentistry of the University of São Paulo (USP, São Paulo, SP, Brazil) and referred to the Special Laboratory of Lasers in Dentistry (LELO) for pain treatment.

#### Exclusion criteria

Participants will be excluded in cases of: congenital problems with involvement of the TMJ and/or orofacial and cervical skull region; neoplastic conditions; history of recent trauma at the orofacial/cervical skull region; use of any type of TMD treatment plaques; functional orthopedic appliances or fixed and/or removable orthodontic appliances; syndromes; cleft lip and/or palatine; psychiatric disorders; severe heart problems; a tooth in severely precarious conditions, such as periodontitis and/or indication for endodontic treatment; those making use of topical or systemic photosensitizing medications; pregnant women; and dermatological diseases in the region where irradiation will be performed.

### Sample calculation

The sample size was calculated based on the main outcome and assuming a Type I error of 5% (significance level), a Type II error of 20% (80% test power), and 50% of magnitude of effect among groups. The total number of participants will be 200 individuals [17, 18], who will be allocated to two groups (active or placebo).

When concluding the treatment of all 200 subjects, researchers will consider the possibility of including more participants (increasing the sample size) to compensate for possible drop-outs. If necessary, the inclusion will be made by adding a new block with participants of both experimental groups (PBMT and placebo) randomly assigned to each of the proposed therapies.

### Intervention

Subjects will be informed about the research and, if they become participants, they will be interviewed, examined, and be submitted to treatment according to the group to which they will be allocated (according to previous randomization). The participants will have the skin cleaned prior to irradiation. All biosecurity precautions will be taken.

All participants will be examined and the trigger points of the pain will be identified, if any. In Group 1 (G1; active treatment, PBMT), the laser will be applied at predetermined points and at specific trigger points identified during clinical examination. The application of the laser will be symmetrical, i.e., on both sides of the face, with the same number of points, irrespective of whether it is a “trigger point” or not. In Group 2 (G2; placebo), the irradiation will be performed as in G1; however, there will be no laser light emission through the tip. The orofacial/cervical skull clinical examination will be performed by one of the researchers (FRC and RQB) who will be blinded to the group in which the participant is taking part. Two items of laser equipment will be used, one for the placebo group and one for the experimental group. The two items of equipment will be labeled with different letters (A and B) and only the researcher in charge of the randomization (PMF) will have access to this information. The researchers (FRC and RQB) who will conduct the laser (placebo or active) application will not be informed of which laser equipment is the active or the placebo type.

The characteristics of the low-power laser equipment and the parameters considered for irradiation are depicted in Table 1.

**Table 1:** Characteristics of the low-power laser equipment and the parameters considered for irradiation

Parameters	
Wavelength (nm)	808
Power (mW)	100
Density of energy (J/cm^2^)	105
Output spot area (cm^2^)	0.028
Energy/point (J)	3
Number of sessions per week	1
Total number of sessions	3
Duration of treatment (weeks)	2

The predefined areas where the laser will be applied are: 1) temporal muscle, three points (one at the anterior muscle beam, one at the middle muscle beam, and one at the posterior muscle beam); 2) masseter muscle, six points (three points at the origin (zygomatic arch) and three points at the insertion (mandibular angle)); 3) medial pterygoid muscle, one medial point located behind the retro molar trigone; 4) sternocleidomastoid muscle, six points (two at the origin of the muscle, two at the middle portion, and two at the insertion); 5) trigger points for pain, one point for each pain point diagnosed under palpation; and 6) the TMJ, three points (one point at the most posterior part of the TMJ region (the introduction of the laser light should be through the outer ear positioning the beam anteriorly), one at the uppermost portion of the TMJ, and one at the anterior portion of the TMJ).

The number of irradiated points will depend on the extra trigger points identified during the clinical examination. If no extra points are identified, the number of points on each side will correspond to 19 points (bilaterally), resulting in 38 points of irradiation per participant.

#### Outcome measure

**Fig. 2:**
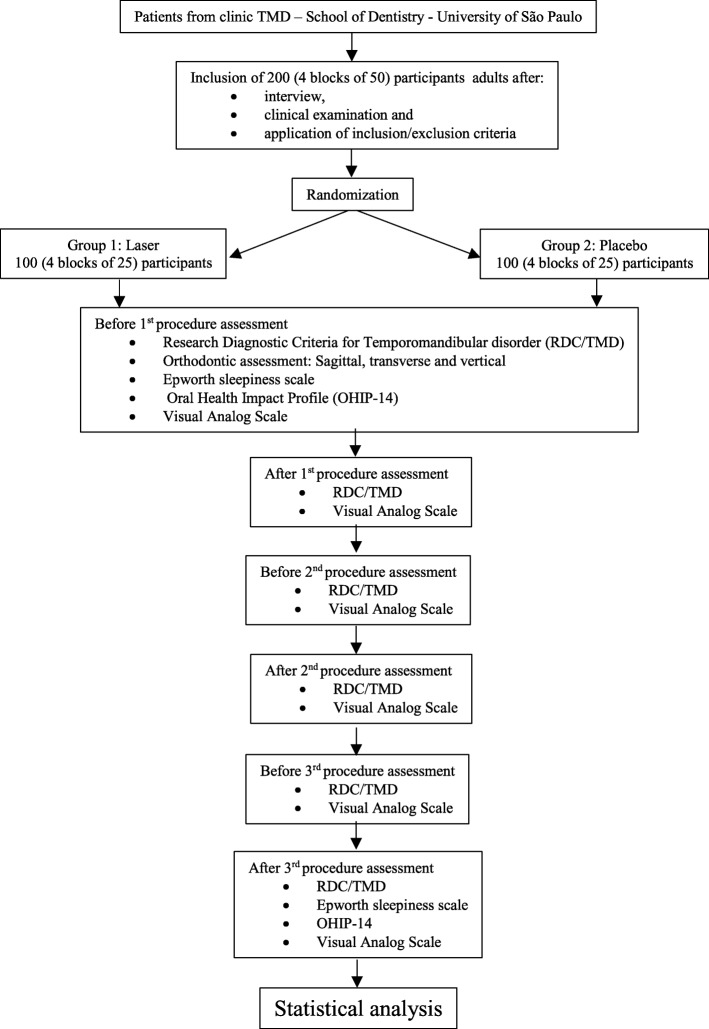
Participant flow diagram. TMD temporomandibular disorder

The schedule of the assessments is described in Fig. 2.

### Assessment of temporomandibular disorders

After the randomization procedure, each participant will be diagnosed using the research diagnostic criteria (RDC) for TMD [19, 20]. The RDC/TMD consists of: palpation of the TMJ and orofacial/cervical muscles; measurement of the mouth opening and mandibular movements using a ruler and caliper; verifying collecting presence and types of articular sounds; and information on parafunctional habits, bruxism, pain, psychological aspects, and chewing. The researchers (FRC and RQB) in charge of applying the questionnaire have experience in the use of this diagnostic instrument, which is considered the gold standard method. However, prior to beginning the study, the researchers (FRC and RQB) will undergo a calibration step, focusing on training for application of the RDC/TMD. Calibration will consist of reading the RDC/TMD manual together [19, 20], discussion of possible doubts, and application of the instrument to one patient, performed by each researcher (FRC and RQB). After checking the results and discussion, should there be any disagreement about the results the researchers will reapply the RDC/TMD form in another participant and, if any discordant points still remain, the form will be applied to other patients for the purpose of calculating the intra- and interobserver Kappa index of agreement [21].

The RDC/TMD assessment will be applied to all participants, before and after each clinical session, by the same researchers (FRC or RQB).

### Orthodontic assessment

The evaluation of occlusion is divided into sagittal analysis, vertical analysis, and transverse analysis [21, 22]. All definitions of the types of occlusions evaluated and how the analysis was divided have been described in a previous study [21]. The researcher (FRC or RQB) will assess the participant’s occlusion before the first clinical appointment.

### Pain assessment

Pain will be assessed using a visual analogue scale (VAS) which consists of a scale (numbered 0–10) similar to a ruler with a line, on which the participant will attribute a grade to his/her pain. Pain “0” represents “no pain” and “10”, the maximum supportable pain. Each participant will receive a diary with a printed VAS to register the pain level throughout the days following the first intervention (7 days). At each clinical appointment, the participant will return this sheet to the researcher (FRC or RQB) and receive a new sheet for the next week.

### Assessment of sleep quality

Painful orofacial and TMJ conditions can influence sleep quality [23]. In this research, the perception of sleep quality will be evaluated using the Epworth scale [24], consisting of questions related to everyday situations that are inadequate due to poor sleep. The scale evaluates the participant’s degree of possibility of sleeping and/or napping. Excessive daytime sleepiness will be assessed with the total score obtained on this scale. This evaluation will be done at the beginning and at the end of the experimental phase, and each participant will fill out his/her own scale.

### Quality of life assessment

The impact of PBMT on participants’ quality of life will be measured. This is a concept used to measure the impact of health conditions on the different dimensions of human life as well as the impact of different therapeutic procedures on patients’ lives.

A specific questionnaire will be used, which will provide numerical scores that can be used to compare groups that were submitted to the two different protocols (active group or placebo) showing the clinical effectiveness of the proposed therapies. The Oral Health Impact Profile (OHIP)-14, validated for the Portuguese language of Brazil, will be used [25]. It measures the discomfort, dysfunction, and self-perceived impact of oral diseases on the daily activities of adults and the elderly [25]. The questionnaire consists of 14 questions. The answers are given according to a scale: 0 = never, 1 = rarely, 2 = sometimes, 3 = often, and 4 = always. The higher the value attributed by the participant the worse the self-perceived impact [25, 26]. The questionnaire will be applied in a quiet environment, without a time limit, in the presence of a single trained researcher (FRC or RQB) [25]. This evaluation will be done at the beginning and at the end of the experimental phase and each participant will fill out his/her own questionnaire.

### Statistical analysis

Before performing the statistical analysis the data will be entered into a spreadsheet and randomly checked by one of the authors (who was not involved in inserting the data into the spreadsheet) to verify whether any error occurred in transcription of the data.

Descriptive statistics will be used to present the characteristics of the participants. Demographic data and clinical characteristics will be compared using the Wilcoxon test or the *t* test for continuous variables; for the categorical variables, the Fisher’s exact or Chi-square test will be performed.

All results will be analyzed using 95% confidence intervals, with a significance level of 5%. The data of the results will also be analyzed by an intention-to-treat analysis (ITT). No interim analyses will be performed since this trial will consist of a short time interval of intervention for each participant.

### Monitoring

There are no reports on the side effects of PBMT. Therefore, there is no intention to unblind or change participants from the groups to which they were allocated. If necessary, the researchers will analyze each case and circumstance to reach a decision. The participant will be informed about this decision.

The researchers expect to improve the participant’s adherence to the study by reminding them of the benefits that PBMT could produce in TMD.

## Discussion

TMD has a direct impact on patient quality of life because it is commonly related to pain and loss of quality of the stomatognathic system functions [27].

PBMT using low-power lasers has been widely indicated for the management of musculoskeletal disorders due to its analgesic, anti-inflammatory, and regenerative effects, and because it is a noninvasive therapy without side effects [11–13].

The effectiveness of PBMT has previously been reported by several authors; however, there is still a lack of controlled clinical trials with methodological quality that could support the benefits of this therapy for temporomandibular dysfunction and that could provide information on the ideal parameters to be used to achieve the desired goals.

This study will be a triple-blinded, randomized controlled clinical trial, the main characteristics of which are the large number of participants and the methodological quality. The main objective will be to investigate whether PBMT is effective in the palliative care of TMD and orofacial/cervical skull pain when using the proposed protocol. On conclusion of this study, the authors expect that some questions regarding the role of PBMT for TMD may be clarified.

## Trials status

This protocol is version 1 and was approved on 14 October 2016. This study is currently ongoing. Recruitment began on 24 October 2016.

## Additional file

Additional file 1: SPIRIT checklist. (DOCX 60 kb)

## Abbreviations

LELO: Special Laboratory of Lasers in Dentistry
OHIP: Oral Health Impact Profile
PBMT: Photobiomodulation therapy
RDC: Research diagnostic criteria
SPIRIT: Standard Protocol Items: Recommendations for Interventional Trials
TMD: Temporomandibular disorder
TMJ: Temporomandibular joint
USP: University of São Paulo
VAS: Visual analogue scale
